# Structural, Antigenic, and Evolutionary Characterizations of the Envelope Protein of Newly Emerging Duck Tembusu Virus

**DOI:** 10.1371/journal.pone.0071319

**Published:** 2013-08-22

**Authors:** Kexiang Yu, Zhi-Zhang Sheng, Bing Huang, Xiuli Ma, Yufeng Li, Xiaoyuan Yuan, Zhuoming Qin, Dan Wang, Suvobrata Chakravarty, Feng Li, Minxun Song, Huaichang Sun

**Affiliations:** 1 College of Veterinary Medicine, Yangzhou University, Yangzhou, China; 2 Institute of Poultry Science, Shandong Academy of Agricultural Sciences, Jinan, China; 3 Department of Chemistry and Biochemistry, South Dakota State University, Brookings, South Dakota, United States of America; 4 Department of Health and Nutrition Sciences, South Dakota State University, Brookings, South Dakota, United States of America; 5 Department of Veterinary and Biomedical Sciences, South Dakota State University, Brookings, South Dakota, United States of America; 6 Department of Biology and Microbiology, South Dakota State University, Brookings, South Dakota, United States of America; Lady Davis Institute for Medical Research, Canada

## Abstract

Since the first reported cases of ducks infected with a previously unknown flavivirus in eastern China in April 2010, the virus, provisionally designated Duck Tembusu Virus (DTMUV), has spread widely in domestic ducks in China and caused significant economic losses to poultry industry. In this study, we examined in detail structural, antigenic, and evolutionary properties of envelope (E) proteins of six DTMUV isolates spanning 2010–2012, each being isolated from individual farms with different geographical locations where disease outbreaks were documented. Structural analysis showed that E proteins of DTMUV and its closely related flavivirus (Japanese Encephalitis Virus) shared a conserved array of predicted functional domains and motifs. Among the six DTMUV strains, mutations were observed only at thirteen amino acid positions across three separate domains of the E protein. Interestingly, these genetic polymorphisms resulted in no detectable change in viral neutralization properties as demonstrated in a serum neutralization assay. Furthermore, phylogenetic analysis of the nucleotide sequences of the E proteins showed that viruses evolved into two distinct genotypes, termed as DTMUV.I and DTMUV.II, with II emerging as the dominant genotype. New findings described here shall give insights into the antigenicity and evolution of this new pathogen and provide guidance for further functional studies of the E protein for which no effective vaccine has yet been developed.

## Introduction

In April 2010, there was a severe disease outbreak in duck farms of the major duck-producing regions in eastern China [1]. The widespread disease affected both meat and laying ducks. The diseased layer ducks had a significant reduction in egg production ranging from 20% to 60%, even up to 90% in some reported cases. Mortality rate could range from 10 to 30%. With disease progression, infected ducks developed some nervous system disorders including unsteady standing, falling and quivering. Similar neurological symptoms were observed in meat ducks, especially 20–40 days old ducklings. Recent studies indicated that domestic chickens were susceptible to this emerging disease with similar symptoms [1], [2], [3], [4], [5]. The new disease has become one of the most economically important infectious diseases of ducks in China [6].

In subsequent studies by several laboratories including our group, the diseased ducks were found to be infected with a new flavivirus [1], [2], [6]. The virus is more closely related to Tembusu virus in the genomic sequence than to other members of flaviviruses, and is therefore provisionally designated duck Tembusu virus (DTMUV). Tembusu virus was first isolated from mosquitoes of the genus Cules in 1955 in Malaysia [7], but the disease outbreak resulting from a mosquito-borne Tembusu virus has not been reported previously in duck [8]. However, it was found that Sitiawan virus, a new strain of Tembusu virus, could cause encephalitis and retarded growth in broiler chicks [8] with clinical symptoms similar to those observed in ducks infected by DTMUV.

As with other flaviviruses [9], DTMUV has a single-stranded and positive-sense RNA genome with approximately 11 kb in length. In virus-infected cell, its genome in the following order: Capsid-prM-Envelop-NS1-NS2A-NS2B-NS3-NS4A-2K-NS4B-NS5 flanked by 5′ and 3′ non-coding regions, is translated into a single polyprotein precursor. Viral and cellular proteases act in concert to cleave the viral polyprotein into three structural and seven non-structural proteins. By analogy with well-studied flaviviruses, the envelope (E) protein of DTMUV is the major surface protein of the virion that mediates binding to the cellular receptor and subsequent fusion event between viral and host membranes [9]. Similar to the E proteins in other flaviviruses, DTMUV E is the primary target of neutralizing antibodies [4]. The E protein of flaviviruses exists as homodimer on the viral membrane that consists of three separate structural domains (I, II, and III) [10]. Domain I (DI) adopts a β–barrel structure and acts as a bridge-like hinge linking the extended domain II (DII) and the globular domain III (DIII) together. DII is formed by two segments that project from loops of DI. The large segment of DII, which is composed of twisted β strands, is stabilized by disulfide bonds and contains a highly conserved hydrophobic fusion loop at its tip. DIII is located at the C-terminus of the E protein and exhibits an immunoglobulin-like structure stabilized by disulfide bonds. Each domain has been assigned with numerous but distinct functions in support of the replication and pathogenesis of flaviviruses [9], [10]. For example, DII and DIII of the E protein are responsible for interaction with the cellular receptor. DII is also essential for anti-parallel homodimerization of the E protein and any mutations in this domain will inhibit viral replication and reduce virulence. As a central unit of E protein complex, DI likely stabilizes the overall orientation of the domains of the E protein. The hinges linking DI and the other two domains, especially histidines in these hinges, are important triggers for conformation change of DII and DIII upon pH change in viral and host cell membrane fusion and homodimer-formation/dissociation processes. Functional antibody and epitope mapping studies demonstrated that all three domains are antigenic and recognized by neutralizing antibodies that inhibit viral entry process, including viral attachment to host cell surface and fusion between viral and host membranes [11], [12], [13]. Numerous studies have shown that many of the most potent neutralizing antibodies target DIII of the E protein probably due to its primary role in binding to the cellular receptor [11], [14], [15].

After the initial nationwide outbreaks in April 2010, no major epidemics of duck Tembusu virus infection were observed throughout 2011 in China and the disease occurred only sporadically in some individual farms. However, in 2012 disease epidemics broke out again in ducks in the major duck-producing region of China. Reason for resurgence of another epidemic just two years apart since its first report is unknown. As with all RNA viruses, the RNA-dependent RNA polymerase of DTMUV may lack a 3′–5′ exonuclease proofreading function for timely correcting replication errors such as removal of misincorporated bases. Replication is therefore error-prone, producing numerous mutations in viral genome. Genetic variability can cause antigenic changes that in turn facilitate the evasion of DTMUV to pre-existing immunity. In addition, selective pressure from host immune system is another force driving viral gene evolution particular the E gene so that the genetic changes can render viruses resistant to anti-E neutralizing antibodies.

To better understand the genetic and antigenic changes of DTMUV isolates and to determine whether antigenic variation plays a role in the recurrent nationwide disease epidemics, we conducted a comprehensive study to investigate structural, antigenic, and evolutionary properties of the E proteins derived from six virus isolates spanning 2010–2012 epidemics, each being obtained from individual farms with different geographical locations where disease outbreaks were reported. We focused on the E protein because it is a major viral envelope protein that frequently undergoes genetic changes due to the evolutionary pressure from host immune system and is the principle target of neutralizing antibodies. The recently resolved crystal structure of the Japanese encephalitis virus (JEV) E protein that shares approximately 65% homology with DTMUV's E serves as a template for our structural modeling work.

Our results showed that six viruses shared about 98.6% identity in their E protein amino acid sequences. The thirteen positions with amino acid variation were found, which were dispersed in the three domains of the E protein. However, these genetic mutations had no detectable effects on neutralizing properties of viruses because similar levels of cross-reactivity were observed among them in a serum neutralization test. Despite the observed cross-reactivity, phylogenetic analysis suggested that two distinct genotypes (DTMUV.I and DTMUV.II) were formed with DTMUV.II emerging as the dominant strain. This result highlights a need for regular surveillance of field strains for the identification of potential genetic mutations that may render viruses escape pre-existing immunity and cause new epidemics. New findings described in this study provide a framework for future functional studies of E protein of this new pathogen toward an effective vaccine that protects poultry health.

## Materials and Methods

### Ethics Statement

Duck infection studies were performed at Shandong Poultry Institute, China and were approved by the Institutional Animal Care and Use Committee of Shandong Poultry Institute (approval number 12–001) and were performed under biosafety level 2+ conditions.

### Virus isolation and RNA extraction

**Figure 1:**
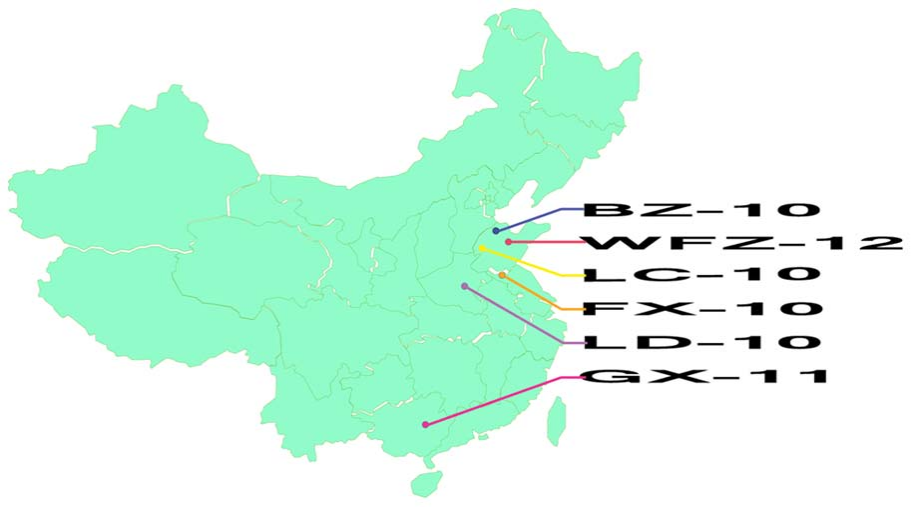
Geographical location of poultry farms from which six virus isolates were obtained.

Clinical samples were collected from six poultry farms that experienced disease outbreaks from 2010 to 2012. Three farms are located in three different provinces, while the other three farms are within one province but have different geographical locations. This information was summarized in Table 1 and Figure 1. Brain or ovary tissues from the diseased ducks were homogenized in sterile phosphate-buffered saline(PBS, pH 7.2)to form a 20% suspension(w/v. After centrifugation at 8000×g for 30 min, the supernatants were filtered by 0.2 µm-pore-size syringe-driven filters. The filtered suspensions were then inoculated into 10-day-old healthy DTMUV antibody-free duck embryonic eggs via allantochorion. Embryonic eggs were examined daily, and the allantoic fluids of dead embryonic eggs were collected after one more passage. The viral RNA was extracted from the allantoic fluids with the MiniBEST viral RNA Extraction kit(TaKaRa).

**Table 1:** Summary of clinical viral isolates.

Strain name	Host	Isolated province	Isolated year
BZ-10	Laying duck	Shandong	2010
LC-10	Meat duck	Shandong	2010
LD-10	Laying duck	Henan	2010
GX-11	Laying duck	Guangxi	2011
FX-12	Laying duck	Jiangsu	2012
WFZ-12	Laying duck	Shandong	2012

### Virus genome amplification and complete genome sequencing

Gene fragments of DTMUV isolate(BZ-10)were firstly amplified with random primers by RT-PCR. Two cDNA fragments corresponding to sizes of 721 nt and 502 nt respectively were obtained and sequenced. Subsequent BLAST analysis revealed that sequence of the 721 nt cDNA fragment had a higher homology with NS5 gene of Tembusu virus(AB110488)and Sitiawan virus(AB026994), reaching to 87.6% and 87.1% homologies, respectively. The 502 nt fragment was more related to E genes of Bagaza virus (AY632545) and Japanese encephalitis virus(U15763) and their corresponding sequence identities were 70.1% and 60.2%, respectively. According to these initial sequence information of DTMUV BZ-10 and full-genome sequences of its closely related viruses, Bagaza virus(AY632545)and Japanese encephalitis virus (U15763), five pairs of overlapping primers (Table S1) were designed to amplify the middle region of BZ-10 genome including the portion of capsid, prM, Envelop, NS1, NS2A, NS2B, NS3, NS4A, 2K, and NS4B-NS5. Both the 5′-end and the 3′end of the genome were amplified using 5′- and 3′-end Full RACE kits(TAKARA). All the PCR products were purified and cloned into pMD18-T vector(TAKARA)for sequencing. LaserGene program (DNAStar) was used to assemble the complete full-length genome sequence of BZ-10 isolate.

Based on the sequence of BZ-10, another five pairs of overlapping primers (Table S2) were designed to amplify and sequence other five DTMUV isolates(LC-10, LD-10, GX-11, FX-12, WFZ-12). Similarly, 5′- and 3′-end Full RACE reactions were performed to determine the 5′- and 3′-end sequences of these five viruses. It should be noted that primer sequences selected are completely identical between BZ-10 and its closely related Tembusu and Sitiawan viruses. We used the traditional Sanger sequencing method and all the derived sequences were the consensus sequences of viral genomes. The genome sequences of six described virus isolates were submitted to Genbank under accession no. KC990540- KC990545.

### Envelope protein structure modeling and Glycosylation site prediction

The structure of the envelope protein of FX-12, a DTMUV isolated in 2012 disease epidemics, was modeled using Modeller. The crystal structure of Japanese Encephalitis Virus (PDBID: 3P54) was chosen as a model template [10]. The N-glycosylation sites of E protein were predicted using Glycam (http://glycam.ccrc.uga.edu). Glycam identifies the potential N-glycosylation sites by calculating the solvent accessible surface area of Asn in Asn-X-Thr/Ser/Cys motifs (X represents any amino acid). For illustration purpose, a β-D-Manp-(1-4)-β-D-GlcpNAc was added to each of the potential glycosylation sites.

### Evolutionary analysis of the E protein

MEGA5 was used to find optimal nucleotide substitution model. The TN93+G (Hasegawa-Kishino-Yano) model was predicted to be the best to describe the substitution patterns for the nucleotide sequences of E genes. Six complete sequences of DTMUV E genes described in this study and other fifteen complete E sequences reported previously [1], [2], [3], [4], [5], [16]with total 21 were used in this study. Viruses that derived these E genes were obtained from diseased meat and laying ducks in affected areas during epidemics spanning 2010–2012. The phylogenetic tree and evolutionary rate were inferred using Bayesian method in Beast [17] (http://beast.bio.ed.ac.uk/Main_Page). Lognormal relaxed uncorrelated molecular clock model was chosen to estimate evolutionary rate; the tree prior was set to a constant size coalescent; the evolutionary rate was calculated separately for the three-codon positions; a uniform distribution was specified as a prior for relative mutation rate sampling. To achieve convergence and derive sufficient effective sample size (>300), 5×10^8^ Markov Chain Monte Carlo iterations were run and trees were sampled every 2000 iterations. The first 10% trees were used as burn-in for tree construction and parameter estimation. Evolutionary rate and divergence time were estimated using Tracer (http://beast.bio.ed.ac.uk/Tracer). Maximum clade credibility trees were constructed using TreeAnnotator and viewed in FigTree (http://beast.bio.ed.ac.uk/FigTree).

### Serum neutralization assay

To generate antisera for serum neutralization, we selected two different DTMUV strains, BZ-10 isolated in the 2010 epidemic and FX-12 isolated in the 2012 epidemic, for inoculation of 7 days healthy DTMUV antibody-negative ducks by hypodermic injection. Inoculated dose was 10^4^ ELD_50_ each duck. Antisera were collected from the ducks 15 days after inoculation. The heat-inactivated antisera, four-fold serially diluted, were respectively mixed with 200 ELD_50_ of each of the six DTMUV strains including two virus isolates (BZ-10 and FX-12) that derived antisera. After the mixture incubated at 37°C for 1 h, they were inoculated into 10-day-old SPF chicken embryonic eggs via allantochorion. Embryonic eggs were checked daily, and dead embryos were recorded up to 6 days post-inoculated. Neutralization titer was determined as the reciprocal of the log2 of the highest dilution that inhibited 50% embryos death. Antibody titers were calculated with the Reed and Muench formula. Each dilution was tested in five embryos. The titers are the mean values of two repeated experiments.

## Results

### Virus isolates and disease outbreaks

To study viral genetic and antigenic changes during 2010–2012 disease epidemics, we isolated six DTMUV strains, each from individual farms where disease outbreaks were documented. As summarized in Table 1 and Fig. 1, two of three 2010 isolates were obtained from two duck farms that are located more than 100 miles away within Shandong province, while the third virus was isolated from a farm in Henan province, located on west of Shandong province. Only one 2011 DTMUV strain was used for this study and the virus was isolated from a diseased duck farm located in Guangxi, one of southernmost provinces in mainland China that shares a border with southeastern Asian countries. Two 2012 isolates were collected from duck farms located in Shandong and Jiangsu (south of Shandong) provinces. It has been reported that the scale of DTMUV-associated epidemics was temporally different [1], [2], [4], [18]. For example, widespread disease outbreaks were recorded in 2010 and 2012, respectively, which were viewed as two waves of DTMUV epidemics, while sporadic activity was observed in 2011. Irrespective of time and scale of epidemics, these viruses caused similar symptoms in ducks characterized by a drop in egg production and neurological disorders with mortality rates reaching approximately 10% (data not shown).

### Genetic and structural analysis of the E protein

Viruses grown in duck embryonic eggs were purified and RT-PCR reactions were subsequently conducted on RNA extractions. The genomic coding sequences for the E protein of six different viruses were determined and used for genetic and structural analysis.

**Figure 2:**
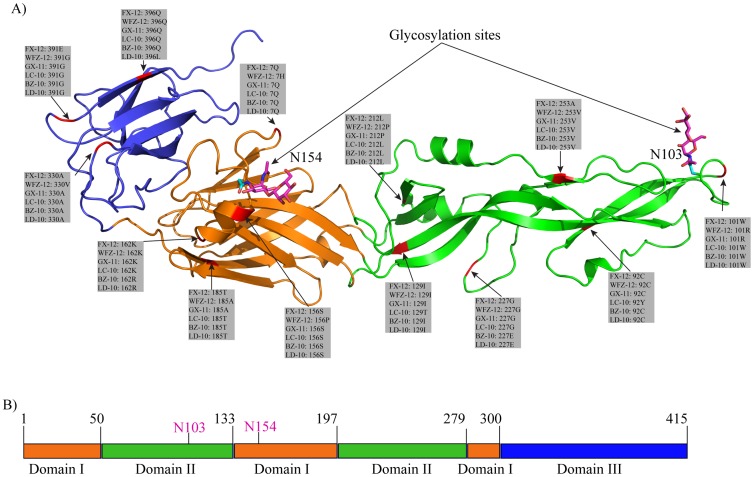
Modeled structure and domain architecture of envelope protein of FX-2012 strain. A) Cartoon scheme view of envelope protein structure. Sites containing amino acid changes were colored red and all these changes were listed in gray boxes. The side-chains of glycosylated residues were colored cyan. For illustration purpose, a β-D-Manp-(1-4)-β-D-GlcpNAc (colored magenta) was added to the glycosylation sites. B). Domain diagram of envelope protein. Domain boundaries were labeled (black) on top of the diagram. The two glycosylation sites were labeled magenta. Domain boundaries were predicted based on domain architecture of Japanese encephalitis virus. Domain colors: orange, domain I; green, domain II; blue, domain III.

**Figure 3:**
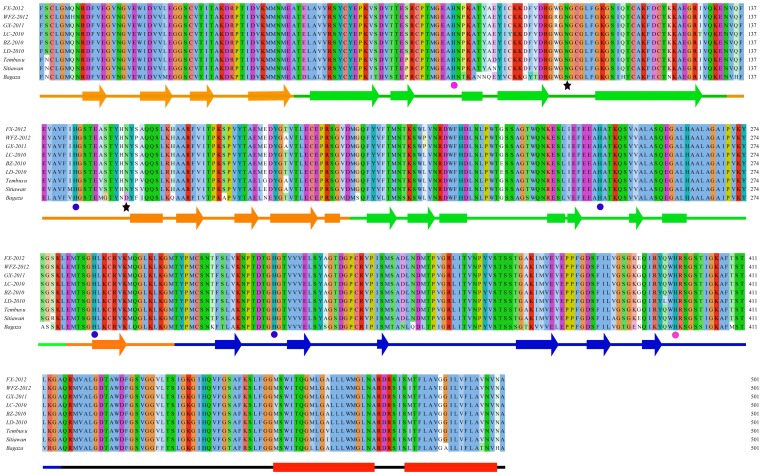
Multiple sequence alignment of the E proteins. The predicted glycosylation sites were marked with star underneath the alignment. Conserved histidines at E protein oligomerization interfaces were labeled with blue circle while histidines conserved in DTMUV and JEV viruses were labeled with magenta circle. The diagram of secondary structure was determined according to the modeled structure of E protein of FX-2012 strain and shown underneath the sequence alignment (rectangle, helix; arrow, β strand). Transmembrane helixes were predicted with TMHMM. Color scheme for sequence alignment position: positive charged amino acids: red; negative charged amino acids, magenta; polar amino acids, green; hydrophobic amino acids, blue. Less conserved positions were colored with lighter color of the corresponding amino acid category. Color scheme for secondary structure: orange, domain I; green, domain II; blue, domain III; red, transmembrane helix.

Multiple sequence alignment demonstrated that six E proteins shared approximately 98% identity (data not shown) and only thirteen positions of the E protein were shown to exhibit genetic polymorphisms (Figs. 2 and 3). To map structurally these mutations in E proteins of six viruses, we took advantage of the recently resolved crystal structure of the E protein of a Japanese Encephalitis virus (JEV) [10], a mosquito-borne zoonotic flavivirus, which has about 65% sequence identity with DTMUV E protein. The relatively high protein identity should allow us to establish a reliable model of DTMUV E protein that can be used to predict its important structural features.

Like most members of the flavivirus, DTMUV has a predicted N-linked glycosylation site at E protein position 154 (Figs. 2 and 3). It has been shown that mutations in this position affected viral infectivity and binding of the cellular receptor [19], [20], [21]. The conservation of this N-linked glycosylation site at the same position in DTMUV suggests that it supports DTMUV replication through a similar mechanism. However, one of the 6 DTMUV strains, WFZ-12 isolated in 2012, has lost this glycosylation site because of a serine to proline substitution at position 156 abolishing the glycosylation of motif (N-X-S/T) (Fig. 3). Curiously, among the three viruses (Tembusu, Sitiawan, and Bagaza) that are closely related to DTMUV, Sitiawan virus that was isolated from diseased chickens, like most of DTMUV strains, maintains this glycosylation site, while two other viruses appeared to lose the glycosylation site at N154 (Fig. 3). Mosquito-borne Tembusu virus resembled WFZ-12 in that despite the presence of asparagine at position 154, position 156 is proline, not serine or threonine, which resulted in a loss of the potential for glycan modification at N^154^. The E protein of Bagaza virus that causes disease in birds has proline, not asparagine, at position 154. Thus, N154 glycosylation site in the E protein is not universally conserved among this group of viruses. Interestingly, the E protein was predicted by Glycam to have an atypical N-linked glycosylation site at position N^103^ (N-X-C) (Fig. 3), which is conserved among all six DMTUV strains and its related viruses (Tembusu, Sitiawan, and Bagaza). The complete conservation of the potential N^103^ glycosylation site suggests an important role of this position in viral infectivity and pathogenesis, which apparently require future investigation.

The E protein of DTMUV possessed four histidines at positions 144, 246, 284, and 319, respectively. By analogy with JEV they were located at the E dimer interface and interdomain (Fig. 3). It is interesting to note that these histidines are completely conserved among E proteins of all flaviviruses. DTMUV E also contained two additional histidines that were also observed in JEV (H^81^ and H^397^) at the same position [10]. These conserved positions have been functionally linked to viral uncoating step in the early stage of flavivirus lifecycle, which is likely orchestrated through protonation of histidines at acidic pH environment [9]. These histidines especially those located at the dimer interface have been also proposed for regulation of E protein oligomerization [10].

### Analysis of genetic variations and antigenic properties of the E proteins of six DTMUV isolates spanning 2010–2012

We next mapped thirteen positions of the E protein showing genetic polymorphisms among six DTMUV isolates using the modeled protein structure. These mutations were located across all three domains of E protein (Figs. 2 and 3). Among them, four and six positions were located in DI and DII, respectively, while three positions were in DIII. Each polymorphism only involved one or two of six analyzed DTMUV viruses (Fig. 2).

Considering that each of three domains is involved in the generation of neutralizing antibody responses against flaviviruses, we next determined the effects of polymorphic mutations on the neutralization sensitivity of these six virus isolates against their homologous and heterologous antisera. We selected two different DTMUV strains, BZ-10 and FX-12, for antibody production in SPF chickens. BZ-10 was isolated in the 2010 epidemic, while FX-12 was isolated in the 2012 epidemic.

**Figure 4:**
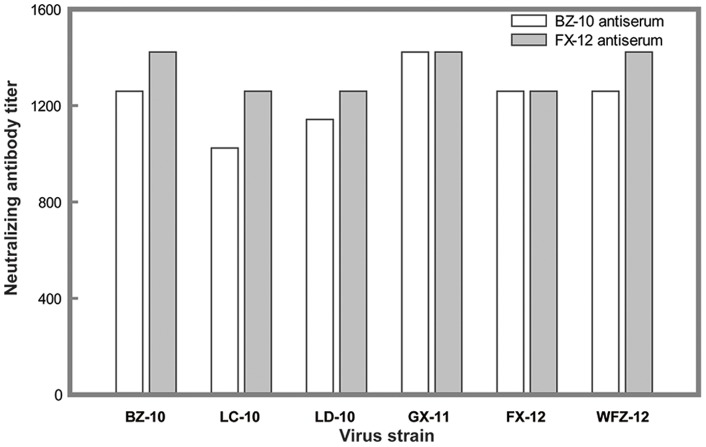
Serum neutralization properties of six DTMUV isolates. Antisera generated from ducks inoculated with BZ-10 and FX-12, respectively, were selected to test the effects of E protein mutations on neutralization sensitivity of each of six viruses as described in Materials and Methods. Each experiment was run in duplicate and repeated twice. The serum neutralizing titers were determined by the minimal serum dilution that inhibited chicken embryo death. The titers were the mean values of two independent experiments.

Antisera generated against BZ-10 an FX-12 neutralized corresponding viruses with high titers 1∶1259 and 1∶1422, respectively. As summarized in Fig. 4, other four viruses were also effectively neutralized by these two antisera with antibody titters ranging from 1∶1024 to 1∶1422, indicating that there was no discernible difference in neutralizing activity among six examined viruses. Thus, results of our experiments suggested that the observed genetic polymorphisms over the past two years in the E protein had no effects on neutralizing properties of these DTMUV strains.

The observation that genetic polymorphisms in the E protein imparted no effects to viral neutralizing property prompted us to perform a direct comparison of DTMUV E-associated amino acid changes to those occurred between Tembusu and Sitiawan viruses (Table 2). It should be noted that little or no cross-neutralization was observed between Tembusu, a mosquito-borne flavivirus, and Sitiawan, a Tembusu-like virus isolated from diseased chickens [8], although only eight amino acid changes were observed between their E proteins. Two of eight mutations are located in hydrophobic helices of the E protein proximal to the membrane (data not shown). Because this region is not linked to stimulation of neutralizing antibodies to flaviviruses, these two mutations were not a major focus of our analysis. Our comparative study was directed to other six mutations that were located across the three domains of the E protein. We reasoned that the direct comparison of E-associated amino acid variations in this group of closely related viruses might give us some insights to critical determinants in conferring neutralization-resistance. As summarized in Table 2, amino acid changes at E positions 156 and 391 are unlikely responsible for little or no cross-neutralization between Tembusu and Sitiawan viruses because these two changes were also present in DTMUV strains and did not alter neutralization property of the virus. As such four other mutations at E positions 89, 143, 277, and 386 that occurred in Tembusu and Sitiawan, but not in all analyzed DTMUV isolates, are likely involved in discriminating Tembusu from Sitiawan in serum neutralization. Interestingly, four mutations were scattered through all three domains of the E protein. Of corresponding positions in DTMUV, three positions had same amino acids as Tembusu and one position was occupied by a unique amino acid differing from both Tembusu and Sitiawan viruses. It will be interesting to evaluate the effects of these mutations on DTMUV's neutralization property in future research.

**Table 2:** Prevalence of the observed polymorphic residues in the E protein between Tembusu and Sitiawan viruses in DTMUV^a^.

Position/E Domain	Tembusu	Sitiawan	DTMUV^b^
89/DII	D	N	E (100)^c^
143/DI	I	M	I (100)
156/DI	P	S	S (96), P (4.0)
277/DII	S	R	S (100)
386/DIII	V	A	V (100)
391/DIII	G	E	G (96), E (4.0)

a E protein sequences were retrieved from the GenBank.

b Prevalence of polymorphic E protein residues observed between Tembusu and Sitiawan viruses in the E proteins of DTMUV (%). Total 25 E proteins were used for analysis.

c Values shown in parentheses indicate prevalence (i.e., %) of amino acid residues of DTMUV in the corresponding positions of the E protein showing sequence variations between Tembusu and Sitiawan viruses.

### Phylogenetic and evolutionary rate analysis

We next sought to determine the evolutionary relationship of E proteins among these six virus isolates. We also incorporated sequence data of E proteins in other DTMUV strains reported previously, JEV, Bagaza, Tembusu and Sitiawan viruses into our analysis [1], [2],[4],[5]. Bayesian phylogenetic trees and estimated divergence time and substitution rate for E proteins of various isolates using their respective nucleotide sequences were conducted.

**Figure 5:**
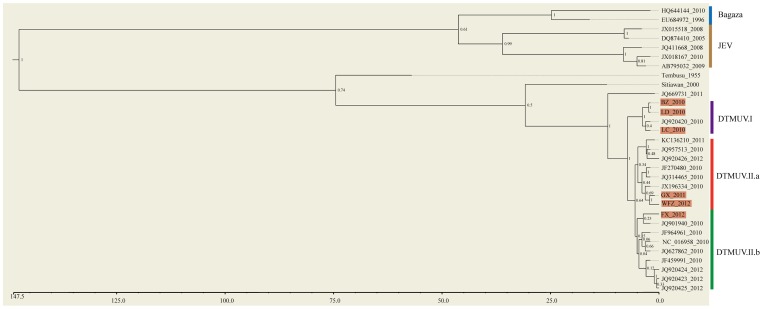
Bayesian phylogenetic tree of viral E proteins. The six strains of DTMUV we sequenced were marked with purple background. The Bayesian posterior probabilities were labeled on each node (1, high confident branching; 0, low confident branching). The bottom scale bar represents divergence time (unit: year).

A phylogenetic tree was inferred from the nucleotide sequences of E proteins (Fig. 5). Overall, two main clusters were observed with Bayesian posterior probability support of 1. One consisted of Bagaza virus and JEV, the other included Tembusu virus, Sitiawan virus and DTMUV. The phylogenetic closeness of DTMUV to the Tembusu and Sitiawan viruses is consistent with results of previous studies [1], [2], [4], [5]. With the exception of one outlier, all DTMUV isolates were clustered into the two clearly defined genotypes (DTMUV.I and DTMUV.II). Interestingly, DTMUV.I had only four strains and all of them were isolated in the 2010 epidemic. DTMUV.II contained sixteen members that were isolated from 2010 to 2012 epidemics. DTMUV.II was further clustered into two subgroups (DTMUV.II.a and DTMUV.II.b), although the posterior probability support was not high (0.64). Both DTMUV.II.a and DTMUV.II.b contained virus isolates ranging from 2010 to 2012. Three of the six viruses we isolated were in group DTMUV.I (BZ_2010, LD_2010 and LC_2010), two in subgroup DTMUV.II.a (GX_2011 and WFZ_2012) and one in subgroup DTMUV.II.b (FX_2012). In summary, our phylogenetic analysis showed that this new duck flavivirus was closely related to Tembusu and Sitiwan viruses. The results also provided preliminary evidence that currently circulating DTMUV strains likely evolved into two independent genetic subgroups. Finally, we estimated the evolutionary rate of this emerging virus. Our data indicated that the estimated substitution rate for DTMUV was about 5×10^−4^ nucleotides per site per year (Fig. S1). This estimation was similar to those reported previously for influenza A viruses [22], indicating that this new pathogen evolved with a relative fast rate.

## Discussion

Previous studies have indicated that DTMUV is more closely related to Tembusu and Sitiawan viruses in Ntaya virus group than to other members of flaviviruses [1], [2], [4], [5]. Our in-depth analysis of viral E proteins confirms this. In addition to the overall sequence and structure similarity, several functional features of the DTMUV E protein are similar to those of well-characterized flaviviruses including Japanese Encephalitis virus (JEV) and Dengue. For example, the DTMUV E protein has four histidines (H^144^, H^246^, H^284^, and H^319^) that are located at the same positions as those of all other flaviviruses, and also possesses two additional histidines at the same positions as JEV E protein [10]. Numerous studies have suggested that these highly conserved histidines play an important role in regulation of E protein oligomerization and promotion of viral entry [23], [24]. However, the reason for the existence and conservation of these two sets of histidines (i.e., completely conserved among all strains and conserved among a subgroup) is still an intriguing question. Because both virus and host components involved in virus entry have been viewed as primary determinants for virus transmission and adaption to a new host species, further investigation of DTMUV E protein-associated histidines may provide novel insights about the evolution and emergence of this new pathogen in duck and potentially in other species. Most E proteins of flaviviruses have a canonical N-linked glycosylation site at position N^154^. This N-linked glycan has been implicated in viral entry, especially in driving the communication between the E protein and the cellular receptor [19], [20], [21]. DTMUV, like most members of flaviviruses, has a potential N-linked glycosylation site at this position. However, one of our DTMUV strains, WFZ-12, and other two related viruses (Tembusu and Bagaza) have lost this site probably during virus evolution. Curiously, all DTMUV, Tembusu and Bagaza viruses appeared to possess a non-canonical glycosylation site at the identical position (N^103^) of their E proteins. Complete conservation of this potential glycosylation is intriguing and suggests its important role in the replication and pathogenesis of these viruses.

The E protein is the major antigenic target of neutralizing antibodies. As with other enveloped RNA viruses such as influenza and HIV, under constant selection pressure from powerful neutralizing antibodies, the major surface glycoprotein such as E in flaviviruses often find a way to evolve genetic mutations or drifts to render viruses escape inhibition. In spite of limited genetic variations that are observed only at thirteen positions compared to 501 amino acids length of DTMUV E, there is a still chance that some of these polymorphisms can alter antigenic property so that DTMUV strains are not cross-recognized against their heterologous neutralizing antibodies. This scenario is best exampled in Tembusu and Sitiawan viruses. Little or no cross-neutralization was demonstrated between these two nearly identical viruses [8], although only eight single mutations are identified in their E proteins. In contrast to Tembusu and Sitiawan viruses, stable antigenicity of DTMUV has been demonstrated in the present work and it suggests that the observed mutations in the E protein so far have not the capacity to cause the antigenic drift. Thus, results of our experiment reveal several interesting points. First, mutations at four positions of the E protein that potentially distinguish Tembusu and Sitiawan viruses antigenically in a serum neutralization assay should be closely monitored in future surveillance program of DTMUV. It is likely that one or these mutations in combination may result in antigenic change of the E protein so that a circulating antigenic variant may emerge and cause new epidemics in ducks. Second, considering that these mutations (between Tembusu and Sitiawan) are not confined exclusively to single domain of the E protein, we speculate by analogy that neutralizing epitopes of this new pathogen localize to regions of the protein that span three separate E domains. Third, some of vaccination strategies that failed to protect poultry health (i.e. recurrence of DTMUV disease outbreaks in vaccinated farms) are probably caused by poor quality of vaccine candidates, not new antigenic variant. Finally, thirteen positions in DTMUV E protein showing genetic polymorphisms seem not to play a direct role in neutralization antibody recognition but may have a role in virus replication, transmission, and pathogenesis, which may warrant future investigation.

It is intriguing to note that the genetic relationship is not always proportional to the time lapse between isolates of DTMUV. For example, some 2010 strains were more related to 2012 or 2011 strains than to other 2010 strains. Consistent with this observation, our phylogenetic tree analysis supports for the presence of two genotypes (DTMUV.I and DTMUV.II). DTMUV.I genotype is relatively small that contains only four 2010 strains. There is the possibility that this small genotype may become gradually extinct, which is being replaced by the large genotype DTMUV.II with increased genetic diversity that can dominate in future disease epidemics. It is intriguing that DTMUV.II genotype contains two emerging subgroups which may diverge since 2010.

DTMUV is an emerging flavivirus that spreads widely in China poultry farms. The disease is highly contagious in ducks and often results a significant reduction in egg production with potentially fatal outcomes. Ecology of this new pathogen is still poorly defined as seasonality of disease outbreaks is not strong and there is little or no evidence showing that this disease is transmitted through mosquitoes. Characteristic studies of structure, antigenicity, and evolution of the E protein presented here shall aid further studies of this new pathogen for poultry as well as provide guidance for future investigation of disease transmission mechanism.

## Supporting Information

Figure S1 **Bayesian phylogenetic tree of viral E proteins.** The six strains of DTMUV we sequenced were marked purple. The evolutionary rates (unit: substitutions per nucleotide site per year) of all branches were shown. The bottom scale bar represents divergence time (unit: year).
(TIFF)

Table S1 **Primers for amplifying BZ-10 genome.**
(DOC)

Table S2 **Primers for amplifying the genomes of the other DTMUV strains.**
(DOC)
