# Supplementary figures and images for: Structural, Antigenic, and Evolutionary Characterizations of the Envelope Protein of Newly Emerging Duck Tembusu Virus
Source: PLoS One. 2013 Aug 22;8(8):e71319. doi: 10.1371/journal.pone.0071319 (PMC3750017; doi:10.1371/journal.pone.0071319)

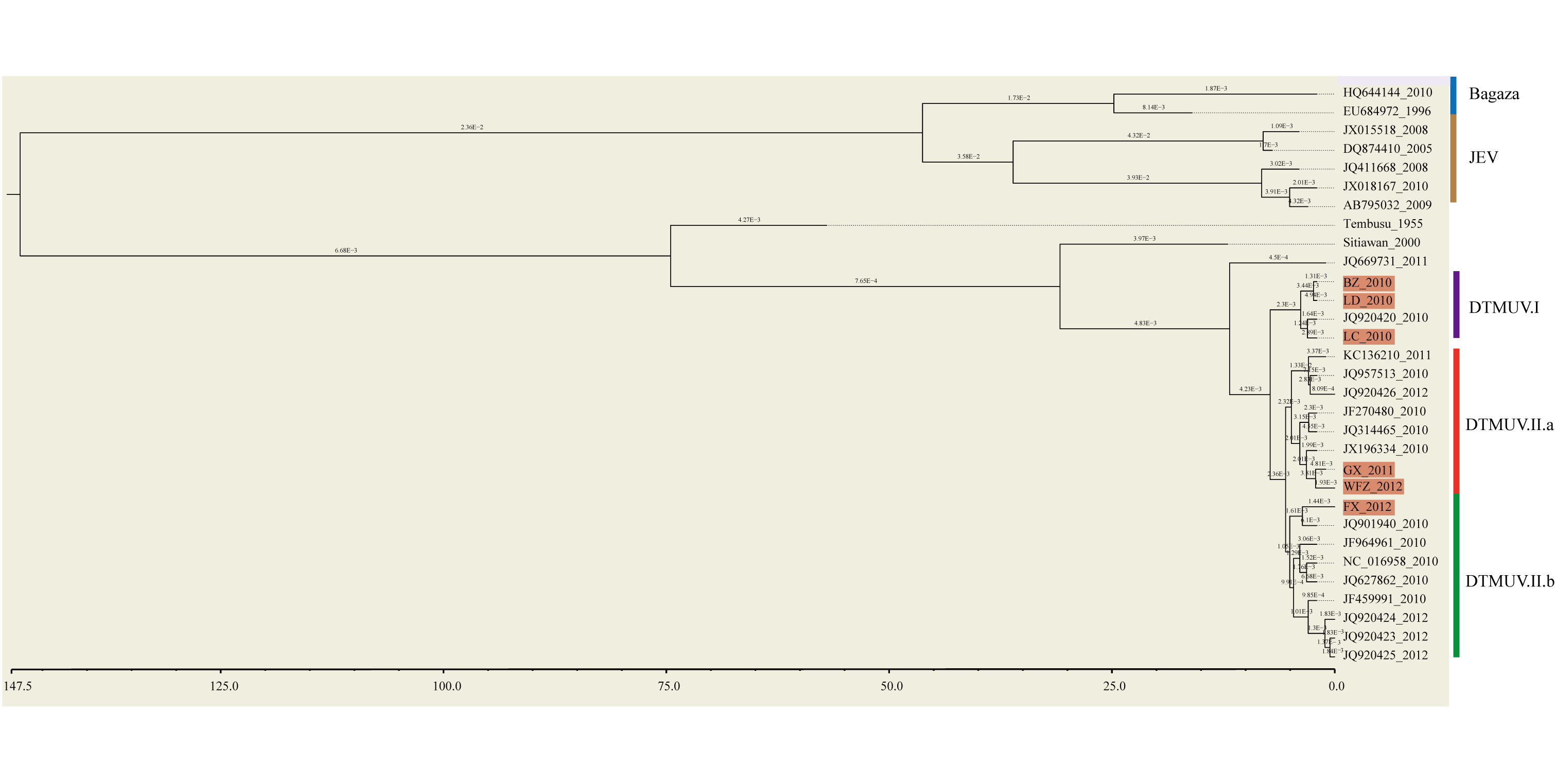

Supplement: Figure S1 — Bayesian phylogenetic tree of viral E proteins. The six strains of DTMUV we sequenced were marked purple. The evolutionary rates (unit: substitutions per nucleotide site per year) of all branches were shown. The bottom scale bar represents divergence time (unit: year). (TIFF) [file pone.0071319.s001.tiff]
