# Supplementary material for: Structural, Antigenic, and Evolutionary Characterizations of the Envelope Protein of Newly Emerging Duck Tembusu Virus
Source: PLoS One. 2013 Aug 22;8(8):e71319. doi: 10.1371/journal.pone.0071319 (PMC3750017; doi:10.1371/journal.pone.0071319)
Supplement: Table S1 — Primers for amplifying BZ-10 genome. (DOC) [file pone.0071319.s002.doc]

**Table S1. Primers for amplifying BZ-10 genome**

| Name | Nucleotide sequence （5’→3’） | Position | Virus＆GenBank accession number |
| --- | --- | --- | --- |
| A-F | AGAAGTTTATCTGTGTGAAC | 1-20 | Bagaza virus/ AY632545 |
| A-R | TGCTCTTCTCTCATCTGGGC | 2886-2905 | DTMUV（BZ-10） |
| B-F | CACATTTGTCATCGATGGACC | 2851-2871 | DTMUV（BZ-10） |
| B-R | TTCACGCTAGGAACAAACCACAC | 5672-5694 | Bagaza virus/ AY632545 |
| C-F | TACAACTTGTTTGTGATGGATGA | 5429-5452 | Bagaza virus/ AY632545 |
| C-R | CCAAAAGGAGTGGTGTCCGTC | 8689-8709 | DTMUV（BZ-10） |
| D-F | GTAAATATGGCCATGACGGAC | 8675-8695 | DTMUV（BZ-10） |
| D-R | GTGACCCAGTCCTCCTGG | 10556-10573 | Bagaza virus/ AY632545 |
| 5U-R1 | CAGCAACTATCGGGAGTAAC | 433-452 | DTMUV（BZ-10） |
| 5U-R2 | GCTTATTCAGTCCGTCGAG | 371-389 | DTMUV（BZ-10） |
| 3U-F | CGGATGTTGGATGACGGTGC | 10493-10512 | DTMUV（BZ-10） |
| 3U-R1 | CGACTAGTTCGTGCAATCCG（T）_20_ |  |  |
| 3U-R2 | ACTAGTTCGTGCAATCCG |  |  |
