# Supplementary material for: Structural, Antigenic, and Evolutionary Characterizations of the Envelope Protein of Newly Emerging Duck Tembusu Virus
Source: PLoS One. 2013 Aug 22;8(8):e71319. doi: 10.1371/journal.pone.0071319 (PMC3750017; doi:10.1371/journal.pone.0071319)
Supplement: Table S2 — Primers for amplifying the genomes of the other DTMUV strains. (DOC) [file pone.0071319.s003.doc]

**Table S2. Primers for amplifying the genomes of the other DTMUV strains**

| Name | Nucleotide sequence （5’→3’） | Position | Virus |
| --- | --- | --- | --- |
| FA-F | ACAGCTTTTGGAGTAGTGCG | 30-49 | DTMUV（BZ-10） |
| FA-R | TGCTCTTCTCTCATCTGGGC | 2886-2905 | DTMUV（BZ-10） |
| FB-F | CACATTTGTCATCGATGGACC | 2851-2871 | DTMUV（BZ-10） |
| FB-R | CCACTCATACCCAGAATTCC | 5622-5641 | DTMUV（BZ-10） |
| FC-F | GGCATTTCCGGACTCCAAC | 5560-5578 | DTMUV（BZ-10） |
| FC-R | TTGTTGTCATTGTGAACTGTCC | 7199-7220 | DTMUV（BZ-10） |
| FD-F | TTGTCAGTACCCCTGCTACTC | 7166-7186 | DTMUV（BZ-10） |
| FD-R | CCAAAAGGAGTGGTGTCCGTC | 8689-8709 | DTMUV（BZ-10） |
| FE-F | GTAAATATGGCCATGACGGAC | 8675-8695 | DTMUV（BZ-10） |
| FE-R | CCAGCTCCATCATTTCAGGC | 10725-10744 | DTMUV（BZ-10） |
| 5U-R1 | CAGCAACTATCGGGAGTAAC | 433-452 | DTMUV（BZ-10） |
| 5U-R2 | GCTTATTCAGTCCGTCGAG | 371-389 | DTMUV（BZ-10） |
| 3U-F | CGGATGTTGGATGACGGTGC | 10493-10512 | DTMUV（BZ-10） |
| 3U-R1 | CGACTAGTTCGTGCAATCCG（T）_20_ |  |  |
| 3U-R2 | ACTAGTTCGTGCAATCCG |  |  |
